# Programmed cell death protein 1 (PD-1) / programmed cell death ligand 1 (PD-L1) in multiple myeloma

**DOI:** 10.1186/s43046-026-00379-2

**Published:** 2026-06-17

**Authors:** Shimaa El-Garf, Reham Hammad, Eman Kandeel, Ahmed Rabea, Mona Alrayes

**Affiliations:** 1 Ain Shams University, Cairo, Egypt; 2 Clinical Pathology Department, Faculty of Medicine (Girls), Al-Azhar University, Cairo, Egypt; 3 National Cancer Institute, Cairo University, Giza, Egypt

**Keywords:** Immune checkpoints, PD-1, PD-L1, Multiple myeloma

## Abstract

**Background:**

The bone marrow microenvironment in multiple myeloma (MM) is characterized by complex immune dysregulation involving multiple inhibitory pathways. Among these, the programmed cell death protein 1 (PD-1)/programmed cell death ligand 1 (PD-L1) axis contributes to T-cell dysfunction; However, its isolated role within this multifactorial network remains incompletely defined.

**Methods:**

This prospective observational study performed a cross-sectional immunophenotypic analysis of 39 newly diagnosed MM (NDMM) patients. PD-1 expression on T cells and PD-L1 expression on bone marrow (BM) plasma cells (PCs) were assessed using flow cytometry. Associations with clinical and laboratory parameters were analyzed using correlation analysis.

**Results:**

PD-L1 mean fluorescence intensity (MFI) on PCs showed a positive correlation with BM T-cell percentage (*r* = 0.350, *p* = 0.029). PD-1 MFI on T cells correlated with total leukocytic count (TLC) (*r* = 0.326, *p* = 0.043). In International Staging System (ISS) stage III patients, PD-L1 expression on PC correlated with T cells% (*r* = 0.501, *p* = 0.018) and TLC (*r* = 0.427, *p* = 0.047), while PD-1 expression showed a strong positive correlation with TLC (*r* = 0.676, *p* = 0.001) and a negative correlation with age (*r* = − 0.492, *p* = 0.020). No significant association was observed with treatment response.

**Conclusion:**

These findings provide an exploratory description of PD-1/PD-L1 expression patterns within the BM microenvironment in MM. Only in advanced cases according to ISS, PD-L1 expression on PC was linked to BM T cells frequency. PD-1/PD-L1 expression patterns were not linked to treatment response.

**Supplementary Information:**

The online version contains supplementary material available at 10.1186/s43046-026-00379-2.

## Introduction

Immune evasion in multiple myeloma (MM) is driven by a multifactorial and redundant network of inhibitory signaling pathways within the bone marrow (BM) microenvironment. Programmed cell death protein 1 (PD-1)/programmed cell death ligand 1 (PD-L1) and other immune checkpoints such as Cytotoxic T-lymphocyte-associated protein 4 (CTLA-4), T-cell immunoglobulin and mucin-domain containing-3 (TIM-3), Lymphocyte activation gene-3 (LAG-3), and T cell immune-receptor with Ig and (immunoreceptor tyrosine-based inhibitory motif) ITIM domains (TIGIT), as well as immunosuppressive populations including regulatory T cells and myeloid-derived suppressor cells, contribute to T-cell dysfunction. Stromal interactions and cytokine-mediated signaling further shape this immunosuppressive niche [1, 2].

Immune checkpoints are involved in tumor progression by playing a critical role in tumor proliferation, angiogenesis, metastasis, chemoresistance, and escaping destruction by the immune system through creating an immunosuppressive environment and avoiding killing by cytotoxic T cells [3]. Within this context, the PD-1/PD-L1 axis represents one component rather than a dominant driver of immune escape, and its isolated evaluation should be interpreted accordingly.

Multiple myeloma is a clonal plasma cell disorder characterized by the accumulation of malignant plasma cells (PCs) in BM, resulting in bone destruction, anemia, renal dysfunction, and hypercalcemia [4]. Despite significant advancements in treatment, including proteasome inhibitors, immunomodulatory agents, and autologous stem cell transplantation, that improve patients’ outcome with increased overall survival (OS), MM remains incurable for most patients due to frequent relapses and drug resistance [5].

Multiple myeloma patients are staged according to the International Staging System (ISS), a widely established and routinely applied risk stratification system based on serum β2-microglobulin and albumin that reflects tumor burden and host status [6].

The PD-1 and its ligand PD-L1 are integral to immune checkpoint mechanisms, maintaining self-tolerance by regulating T-cell activity. In cancers, including MM, these pathways are often exploited to suppress immune responses, facilitating tumor progression [7, 8]. PD-1, expressed on activated T cells, binds to its ligands PD-L1 and PD-L2, which are frequently upregulated in malignancies, leading to T-cell exhaustion and immune evasion [9].

Studies highlight a significant increase in PD-L1 expression on plasma cells and PD-1 expression on T cells within the BM microenvironment [10, 11]. This pathway is further exacerbated by cytokines and inflammatory mediators that modulate PD-L1 expression, creating a permissive niche for myeloma cell growth [7, 9].

Targeting the PD-1/PD-L1 axis using immune checkpoint inhibitors (ICIs) has shown promising outcomes in other cancers. Emerging data in MM suggests potential therapeutic benefits, particularly with other agents to overcome resistance mechanisms. However, their role in MM remains limited, reflecting the complexity of the immune microenvironment [8, 11].

This study was designed as an exploratory immunophenotypic to investigate the expression of PD-1 on BM T cells and PD-L1 on plasma cells in newly diagnosed (NDMM) patients. Additionally, the relationship between PD-1/PD-L1 expression and clinical parameters was evaluated, including disease stage, laboratory findings, and response to first-line chemotherapy.

## Methods

### Study design and participants

This was a prospective observational study with cross-sectional analytical design at the time of diagnosis conducted between November 2021 and December 2023. This study aimed to investigate the expression of PD-1 on BM T cells and PD-L1 on plasma cells in NDMM patients in addition to the relationship between PD-1/PD-L1 expression and clinical parameters. It was evaluated according to disease stage and laboratory findings.

The cases were recruited from the National Cancer Institute, Cairo University, Egypt. A total of 39 patients with NDMM were enrolled in the study prior to therapy, with the mean age was 55.5 years (range 30–77). Diagnosis was established based on International Myeloma Working Group (IMWG) criteria 2016, including clonal plasma cell infiltration in the bone marrow and/or myeloma-defining events (MDE); hypercalcemia, renal insufficiency, anemia, and bone lesions [12]. Patients were categorized according to the ISS to stage I, *n* = 2 (5.1%), stage II, *n* = 15 (38.5%), and stage III, *n* = 22 (56.4%).

### Inclusion criteria were

Adults aged ≥ 18 years, newly diagnosed and treatment-naïve MM patients, and the willingness to participate and provide informed consent. Exclusion criteria were previous chemotherapy or immunotherapy intake and the concurrent active malignancies or autoimmune disorders.

The study was approved by the Research Ethical Committee of the Faculty of Medicine for Girls, Cairo, Al-Azhar University (FMG-IRB) and followed the declaration of Helsinki (IRB number 201909165).

### Flowcytometry and laboratory assessment

#### Sample collection and preparation

Bone marrow aspirates and peripheral blood samples were collected at diagnosis. Bone marrow samples were processed immediately after collection to minimize cellular degradation and pre-analytical variability. Bone marrow sample quality was carefully evaluated to minimize hemodilution bias. Morphological assessment and flow cytometric event distribution were used to confirm representative marrow sampling. Samples with discordant cellular composition suggestive of peripheral blood admixture were excluded from analysis. Peripheral blood samples were used for routine hematological and biochemical laboratory parameters. Clinical data, including demographics and presenting symptoms, were recorded at diagnosis.

#### Hematological and biochemical analysis

Complete blood counts (CBC) were performed on EDTA-anticoagulated blood using an automated Coulter hematology analyzer (Beckman Coulter, Switzerland). Serum creatinine, calcium, uric acid, lactate dehydrogenase (LDH), total proteins, albumin, and β2-microglobulin were measured using a Beckman CX9 auto-analyzer (Beckman Coulter, Switzerland).

Serum protein electrophoresis was performed using semi-automated agarose gel electrophoresis (Sebia systems) to detect monoclonal proteins. Immunofixation (IF) electrophoresis (Hydragel IF, Sebia HYDRASYS) was used to determine immunoglobulin (Ig) heavy and light chain restriction according to manufacturer instructions.

#### Bone marrow morphology and plasma cell assessment

Bone marrow aspirates were evaluated by manual differential count on Leishman-stained smears. Plasma cell burden was recorded as percentage of total nucleated marrow cells.

#### Flow cytometry analysis

Immunophenotyping was performed using a multicolor flow cytometer (Navios EX, Beckman Coulter, Fullerton, CA, USA) on fresh samples. Two independent gating strategies were applied corresponding to each antibody panel based on FSC/SSC for debris exclusion) followed by singlet discrimination (FSC-A vs. FSC-H).

For the T-cell panel, lymphocytes were identified using CD45/ side scatter (CD45/SSC) characteristics, high expressing CD45 + cells with low SS were gated, then T lymphocytes were identified as CD3 + events. PD-1 expression was subsequently assessed within the CD3 + population.

Plasma cells were defined as cells expressing CD38 bright/CD138 + events, and PD-L1 expression was evaluated within this gated population. Representative gating plots are provided in Supplementary Figure S1.

#### Definition of expression data

Two antibody panels were applied per patient: T-cell checkpoint panel: PD1 (CD279)-APC-conjugated antibody (BD Biosciences; San Jose, California, USA, cat. no. A07764), CD3-FITC-conjugated antibody (Immunotech; Beckman Coulter, Marseille, France; cat. no. P59232AA) and CD45-PC5.5-conjugated antibody (Immunotech; Beckman Coulter, Marseille, France; cat. no. A54139). Plasma cell checkpoint panel: CD38- FITC-conjugated antibody (BD Biosciences, USA Cat. no. 560982, lot no. 345766), CD138- PC5-conjugated antibody (BD Biosciences, USA Cat. no. 561704, lot no. 6116778), and PD-L1 (CD274)- APC-conjugated antibody (Life Science; Beckman Coulter, Marseille, France; cat. no B59756-AB, lot no. 200022). Each tube contained 50 µL of sample and 5 µL of antibody cocktail and was incubated according to standardized laboratory protocol.

All patients included in the study were diagnosed MM based on flow cytometric immunophenotyping. Plasma cell clonality was confirmed by the expression of either inter-cytoplasmic kappa or inter-cytoplasmic lambda light chains. Only clonal plasma cells were included in the analysis of PD-L1 expression while normal PC were excluded.

#### Sample adequacy and hemodilution control

Samples showing significant plasma cell count by flowcytometry at time of initial diagnosis were included and were compared to BM film assessed morphologically. Samples showing discrepancy shown as low flow cytometry count compared to morphological assessment of BM film were excluded in subsequent flow cytometric analysis of PD1/ PDL1 axis.

#### Quality control

Instrument performance was verified daily using compensation set and immune-check tubes. Standardized gating templates were applied across all samples. Analyses were independently reviewed by two experienced investigators to ensure reproducibility and reduce interpretative bias.

### Statistical analysis

Data were analyzed using SPSS v25.0. Descriptive statistics were presented as median (range) or mean ± standard deviation. Correlations between PD-1/PD-L1 expression and clinical/laboratory parameters were assessed using Pearson or Spearman correlation tests that were applied according to data distribution. PD-1 and PD-L1 expression were evaluated using both percentage positivity and mean fluorescence intensity (MFI). A p-value < 0.05 was considered statistically significant. Data was analyzed using IBM SPSS Statistics (V. 26.0, IBM Corp., USA, 2019. Variables analyzed included: Age, ISS stage, TLC, T Cells percentage and Laboratory parameters. Performance status and cytogenetic risk stratification (e.g., Fluorescence in situ hybridization (FISH)) were not consistently available and therefore not included in analysis, representing a limitation.

Given the exploratory nature of the study, no correction for multiple comparisons was applied; therefore, subgroup analyses were considered hypothesis-generating and interpreted cautiously due to limited sample size. Also, multivariate analysis was not performed due to sample size limitations.

## Results

### Demographics and clinical characteristics

The study included 39 patients (19 males and 20 females). The mean age of patients was 55.5 years (range: 30–77), with female-to-male ratio of 1.05: 1. Hypercalcemia was present in 20.5% of cases, renal impairment (elevated creatinine) in 38.5% of cases and anemia (Hemoglobin < 10gm/dL) in 69.2% of the patients. Clinical and laboratory data were summarized in Table 1. Lymphadenopathy and hepatosplenomegaly were observed in 23.1% and 28.2% of patients, respectively, based on clinical examination and available imaging.

**Table 1:** Baseline demographic, clinical, and immunochemical characteristics including immunoglobulin isotype and light chain restriction of NDMM in this study (*n*=39)

Demographic parameter	Mean ± SD (range)	
Age (years)	55.5 ± 10.4 (30–77)	
Sex Females *n* (%)	20 (51.3%)	
Males *n* (%)	19 (48.7%)	
Lab/Clinical Parameter	Number (%)	
Hemoglobin (gm/dL) Ref: Male: 13.5–17.5 g/dL, Female: 12.0–15.5 g/dL	< 10	27 (69.2%)
Serum Calcium (mg/dL) Ref (8.5–10.5 mg/dL)	> 11	8 (20.5%)
Serum Creatinine (mg/dL) Ref (0.6–1.2 mg/dL)	> 2	15 (38.5%)
β2 microglobulin (mg/L) Ref (0.7–3.4 mg/L)	High > (3.4 mg/L)	37 (94.9%)
ISS stage details (n)	I	2 (5.1%)
	II	15 (38.5%)
	III	22 (56.4%)
Immunofixation	IgA Kappa	2 (5.1%)
	IgA lambda	6 (15.4%)
	IgG kappa	27 (69.2%)
	IgG lambda	4 (10.3%)
Kappa restriction	Positive	29 (74.4%)
Lambda restriction	Positive	10 (25.6%)
Lymphadenopathy *n* (%)	Positive	9 (23.1%)
Hepatosplenomegaly *n* (%)	Positive	11 (28.2%)
Constitutional symptoms (Fever and weight loss) *n* (%)	Positive	26 (66.7%)

Data are presented as mean +/-standard deviation for continuous variables and as number (percentage) [*n* (%)] for categorical variables

*SD* Standard Deviation, *ISS* International Staging System, *IgA* Immunoglobulin A , *IgG* Immunoglobulin G, *n* Number of Patients, *NDMM* Newly diagnosed multiple myeloma, *Ref* Reference Range

Expression of PD-1/PD-L1: Flow cytometry analysis demonstrated that the median plasma cells in BM was 45% (14–89) and the median PD-1 expression on T cells was 19% (range: 0.2–98.1%) while the median PD-1 MFI on T cells was 2 (range: 0.12- 43.0%). The median PD-L1 expression on plasma cells was 22% (range: 3.1–100%), and the median PD-L1 MFI on plasma cells was 3.4 (range: 0.15- 40), as shown in Table 2.

**Table 2:** PD-L1 and PD-1 expression in the studied MM patients

Parameter	Median (Range)
Bone marrow aspirate plasma cells % Ref (< 5% of ANCs))	45 (14–89)
T cells % Ref (3–8% of ANCs)	11.70 (1.70–23.30)
PD-L1^+^ CD138^+^ CD38^+^ %	22 (3.1–100)
PD-L1 MFI	3.40 (0.15- 40)
PD-1^+^ T cells %	19.00 (0.20–98.10)
PD-1 MFI	2.00 (0.12- 43.00)

Data are presented as median (range)

*ANCs* All Nucleated Cells, *PD-1* Programmed Cell Death Protein 1, *PD-L1* Programmed Cell Death Ligand 1, *CD* Cluster of Differentiation, *MFI* Mean Fluorescence Intensity

### Comparison between groups as regard laboratory results

There was a significant elevation in creatinine level (p 0.012), β2 microglobulin (*p* < 0.001), and uric acid (p 0.019) among patients in stage III in comparison with patients in stage I & II. There was a significant elevation in TLC (p 0.043), and uric acid (p 0.039), among patients in the non-responding group in comparison with patients in the responding group. There was a significant elevation in PD-1^+^ T cells % and serum albumin among patients presenting with normal β2 microglobulin in the comparison with patients with high β2 microglobulin.

### Correlation with clinical parameters

No significant correlation was found between PD-1 or PD-L1 expression in the NDMM with patient characteristics; age, monoclonal types, light chain, and ISS staging in the present study.

**Fig. 1:**
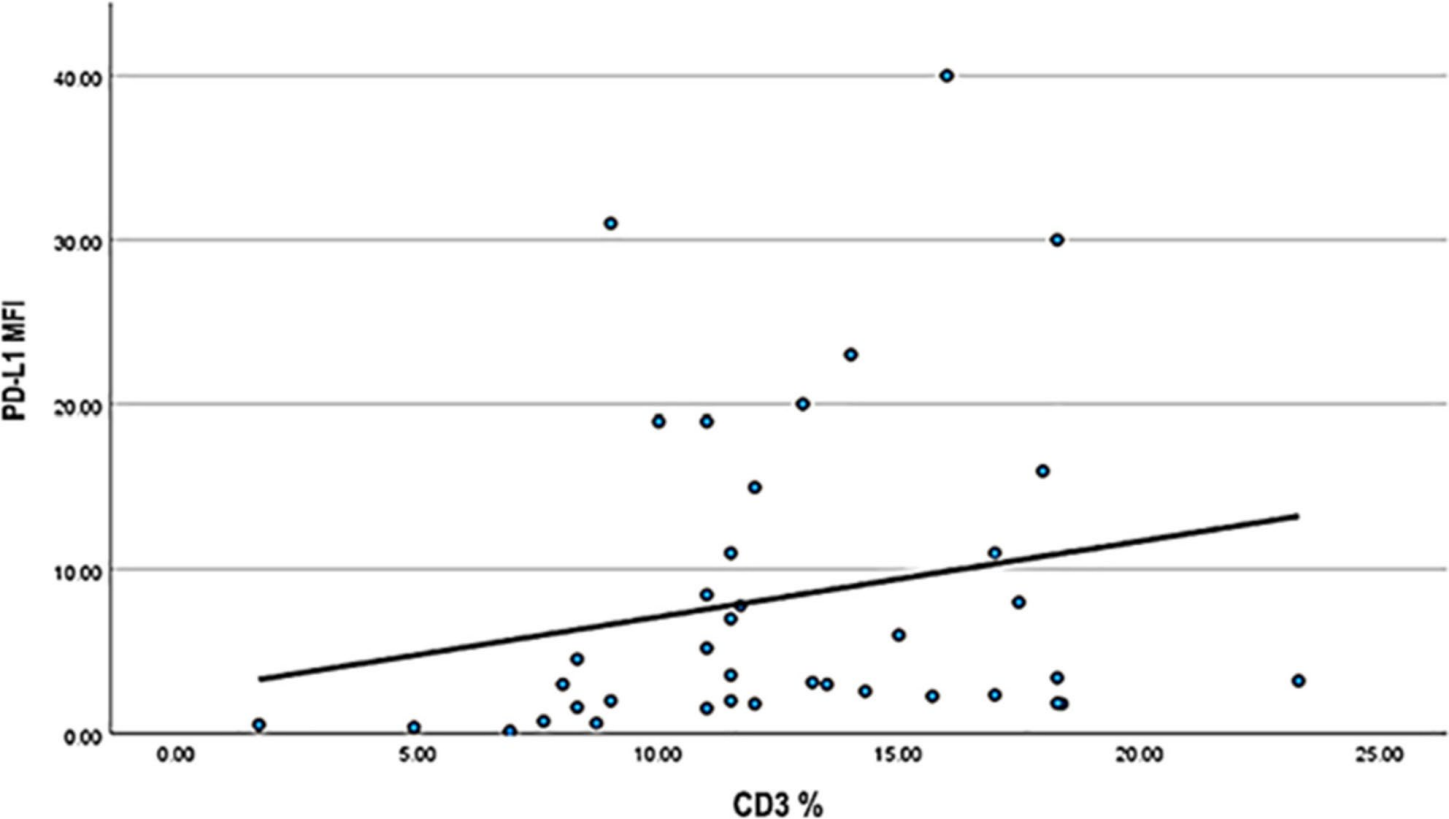
A significant positive correlation between percentage of T cells and PD-L1 density on myeloma cells

**Fig. 2:**
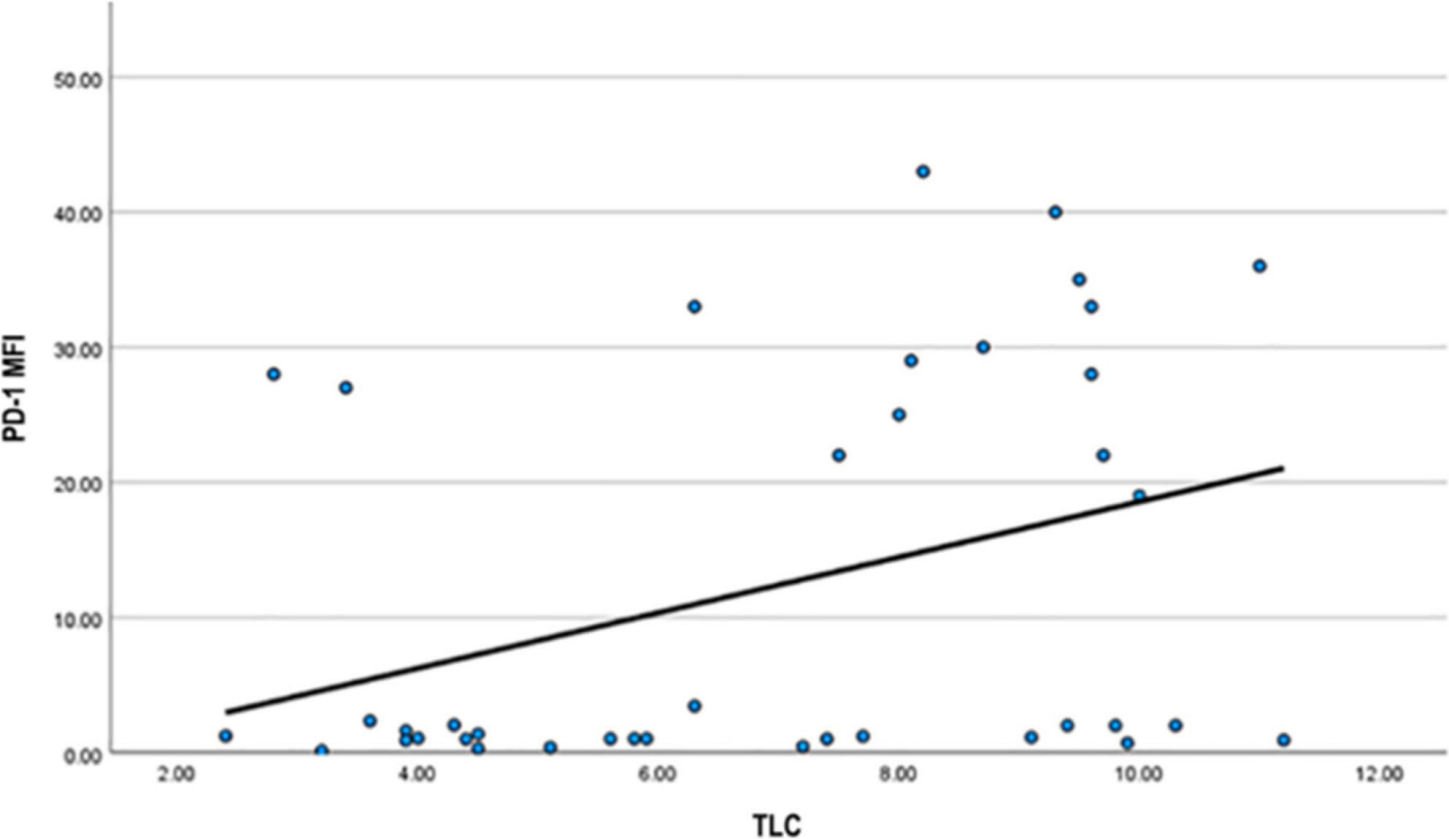
A significant positive correlation between total leucocytic count and PD-1 density on T cells

A significant positive correlation was observed between T-cell percentage and PD-L1 MFI on plasma cells (*r* = 0.350, *p* = 0.029) Fig. 1, as well as between total leukocytic count and PD-1 MFI on T cells (*r* = 0.326, *p* = 0.043) Fig. 2. No significant correlation was detected between total leukocytic count and PD-L1 MFI (*p* = 0.075).

In patients with stage III disease, PD-1 MFI demonstrated a strong positive correlation with total leukocytic count (*r* = 0.676, *p* = 0.001) and a significant negative correlation with age (*r* = -0.492, *p* = 0.020). Similarly, PD-L1 MFI showed significant positive correlations with both T-cell percentage (*r* = 0.501, *p* = 0.018) and total leukocytic count (*r* = 0.427, *p* = 0.047) as shown in (Table 3).

**Table 3:** Correlation of PD-L1 and PD-1 mean fluorescence intensity with clinical and laboratory parameters

Parameters	PD-L1 MFI		PD-1 MFI	
	Correlation Coefficient	*P* value	Correlation Coefficient	*P* value
T cells % (*N* = 39)	0.350	0.029	0.095	NS
Total leukocytic count (×10⁹/L) (*N* = 39)	0.288	NS	0.326	0.043
Age (years), ISS stage III (*N* = 22)	−0.235	NS	−0.492	0.020
T-cell percentage, ISS stage III (*N* = 22)	0.501	0.018	0.193	NS
Total leukocytic count (×10⁹/L) ISS stage III (*N* = 22)	0.427	0.047	0.676	0.001

*PD-1* Programmed Cell Death Protein 1, *PD-L1* Programmed Cell Death Ligand 1, *MFI* Mean Fluorescence Intensity, *TLC* Total Leukocytic Count, *NS* Not Statistically Significant, *N* Number of Patients

*P* values < 0.05 were considered statistically significant. NS = not statistically significant

Using the median PD-L1 MFI value (3.40) as the cutoff, patients were classified into low and high PD-L1 expression groups. No statistically significant differences were observed between the two groups regarding clinical characteristics or laboratory parameters. In contrast, PD-1 MFI on T cells was significantly higher in the high PD-L1 expression group than in the low PD-L1 expression group (*p* < 0.001). This finding suggests a statistical association between PD-L1 expression on plasma cells and PD-1 expression on T cells (Table 4).

**Table 4:** Comparison study using the median of PD-L1 MFI to subgroup into high and low expression groups with laboratory parameters

	**PD-L1 MFI**		***P*** ** value**	**Sig**
**Parameters**	**low expression**	**high expression**		
	**Median (Range)**	**Median (Range)**		
PD-1 MFI	1.04 (0.12- 30)	27 (1.02- 43)	< 0.001	HS

Patients were categorized into low- and high-expression groups according to the median PD-L1 MFI value. Data are presented as median (range)

*PD-1* Programmed Cell Death Protein 1, *PD-L1* Programmed Cell Death Ligand 1, *MFI* Mean Fluorescence Intensity, *HS* Highly Significant

*P* values < 0.05 were considered statistically significant, and *P* values < 0.001 were considered highly significant (HS)

## Discussion

Multiple myeloma accounts for 10–17% of hematologic malignancies worldwide, and its incidence has more than doubled in the past three decades [13]. According to Globocan in Egypt, 2020, an estimated 736 new MM cases in 2018 were found, representing 0.55% of all new cancer cases. The number of MM-related deaths accounts for nearly 649 deaths (0.93% of all deaths) [14].

The PD-1 / PD-L1 pathway is a critical inhibitor of immune activation. The immune checkpoint molecule PD-L1 was reported to be highly expressed on plasma cells. Immunotherapy represents an attractive strategy to achieve durable remissions [15].

Therefore, this study provides an exploratory assessment of PD-1/PD-L1 expression within the BM microenvironment of newly diagnosed MM patients through analyzing the expression of PD-L1 on MM cells and the expression of PD-1 + on T-cells in 39 newly diagnosed MM patients to explore the contribution of the PD-1/PD-L1 axis within the broader immune microenvironment of MM. The level of PD-1^+^ T cells in BM from patients with MM was analyzed in relation to clinical and laboratory parameters. PD-1 expression alone does not distinguish between T-cell activation and exhaustion states, limiting functional interpretation.

In the present study patients were staged as follows: 5.1% stage I, 38.5% stage II and 56.4% stages III respectively. Advanced stage at presentation may reflect delayed presentation or referral patterns, though not specifically evaluated in this study.

Plasma cell infiltration of the liver has been reported in up to 45% of multiple myeloma cases at autopsy and is associated with hepatomegaly and adverse prognosis (Cesar et al., 2019). In the present study, Lymphadenopathy and hepatosplenomegaly were assessed based on clinical examination and available imaging records (ultrasound/ Computed Tomography (CT) where applicable) and were observed in 28.2% and 23.1% of patients, respectively, which is higher than previously reported rates in Egyptian cohorts (Husseiny et al., 2019). These findings may reflect advanced disease biology or coexisting conditions rather than typical MM presentation.

The median PD-L1 expression on plasma cells was 22%, and PD-1 expression on T cells was 19%. While PD-L1 was consistently expressed in myeloma cells, no significant correlations were found between its levels and clinical parameters such as age, monoclonal type, ISS stage, or treatment response. Subgroup analysis also revealed no significant differences between patients with high and low PD-L1 expressions. The lack of strong clinical associations in the present study aligns with the limited efficacy of PD-1 / PD-L1 inhibitors in MM, highlighting the complexity of immune escape mechanisms beyond a single checkpoint axis.

Patients with high PD-L1 expression demonstrated significantly higher PD-1 MFI on T cells compared with patients with low PD-L1 expression (*p* < 0.001), suggesting an association between increased PD-L1 expression on plasma cells and increased PD-1 expression on T cells, although no functional validation was performed. The findings remain descriptive and do not establish functional immune exhaustion or activation.

A notable finding was the significant negative correlation between the age of patients and the PD-1 expression on T cells in stage III and the PD-1 density in stage II, possibly reflecting immune dysfunction and its impact on disease progression and response to immunotherapy. Quan et al. (2023) stated that aging in elderly individuals was accompanied by a decrease in the counts of T-cell subpopulations [16].

Total leucocytic count was positively correlated with PD-1 density that may reflect underlying biological variability in disease and host response.

The study aligns with previous research showing high PD-L1 expression in MM but differs in its prognostic significance, with no association found between PD-L1 levels and tumor burden. Differences in measurement methods and patient populations, small sample size, may explain these inconsistencies.

The limited clinical efficacy of PD-1 inhibitors in MM, as reported in recent studies (PMID: 36212502), underscores that immune escape in MM is driven by a complex and redundant network rather than a single checkpoint pathway [17].

Therapeutically, while immune checkpoint inhibitors targeting PD-1/PD-L1 have shown efficacy in other cancers, their role in MM remains unclear. The lack of significant clinical correlations highlights the complexity of immune evasion in MM and underscores the need for combination therapies. Further research is required to understand the role of PD-1/PD-L1 in MM’s immune microenvironment and explore novel therapeutic approaches targeting this pathway and including relapsed or refractory MM.

The study has limitations, including the relatively small sample size and single-center design, which may limit the generalizability of findings. Additionally, the cross-sectional design limits interpretation of immune dynamics, as checkpoint expression is known to evolve during treatment and disease progression. The absence of functional immune assays limits interpretation of whether observed expression patterns reflect true T-cell exhaustion or reactive immune activation. Also, Cytogenetic and molecular risk stratification data were not available, limiting the ability to correlate immune profiles with genomic risk.

## Conclusion

This study provides an exploratory characterization of PD-1/PD-L1 expression patterns and the immune cell distribution in the bone marrow of MM patients. Only in advanced cases according to ISS, PD-L1 expression on PC was linked to BM T cells frequency. PD-1/PD-L1 expression patterns were not linked to treatment response. Yet, further studies integrating functional, longitudinal, and molecular data are required.

### Recommendations

Further research is needed to extend the study of PD-L1 expression on neoplastic plasma cells in relapsed MM, Smoldering MM (SMM), and Monoclonal Gammopathy of Undetermined Significance (MGUS).

## Supplementary Information

Supplementary Material 1.

## Data Availability

The data that support the findings of this study are not openly available due to reasons of sensitivity and are available from the corresponding author upon reasonable request.

## Abbreviations

BM: Bone Marrow
CBC: Complete Blood Count
CD: Cluster of Differentiation
CT: Computed Tomography
CTLA-4: Cytotoxic T-lymphocyte-associated protein 4
FISH: Fluorescence In Situ Hybridization
FSC: Forward Scatter
ICIs: Immune Checkpoint Inhibitors
IF: Immunofixation
Ig: Immunoglobulin
IMWG: International Myeloma Working Group
ISS: International Staging System
ITIM: Immunoreceptor Tyrosine-Based Inhibitory Motif
LAG-3: Lymphocyte Activation Gene-3
LDH: Lactate Dehydrogenase
MDE: Myeloma-defining Events
MFI: Mean fluorescence Intensity
MGUS: Monoclonal Gammopathy of Undetermined Significance
MM: Multiple Myeloma
NDMM: Newly diagnosed multiple myeloma
OS: Overall Survival
PCs: Plasma Cells
PD-1: Programmed cell death protein 1
PD-L1: programmed cell death ligand 1
SMM: Smoldering MM
SSC: Side Scatter
TIGIT: T-cell Immunoreceptor with Immunoglobulin and ITIM Domains
TIM-3: T-cell Immunoglobulin and Mucin-Domain Containing-3
TLC: Total leucocytic count
