# Supplementary material for: Programmed cell death protein 1 (PD-1) / programmed cell death ligand 1 (PD-L1) in multiple myeloma
Source: J Egypt Natl Canc Inst. 2026 Jun 17;38:35. doi: 10.1186/s43046-026-00379-2 (PMC13313294; doi:10.1186/s43046-026-00379-2)
Supplement: Supplementary file 1 — Supplementary Material 1. [file 43046_2026_379_MOESM1_ESM.docx]

Two antibody panels were applied per patient:

- T-cell checkpoint panel: PD1 (CD279)-APC-conjugated antibody (BD Biosciences; San Jose, California, USA, cat. no. A07764), CD3-FITC-conjugated antibody (Immunotech; Beckman Coulter, Marseille, France; cat. no. P59232AA) and CD45-PC5.5-conjugated antibody (Immunotech; Beckman Coulter, Marseille, France; cat. no. A54139).
- Plasma cell checkpoint panel: CD38- FITC-conjugated antibody (BD Biosciences, USA Cat. no. 560982, lot no. 345766), CD138- PC5-conjugated antibody (BD Biosciences, USA Cat. no. 561704, lot no. 6116778), and PD-L1 (CD274)- APC-conjugated antibody (Life Science; Beckman Coulter, Marseille, France; cat. no B59756-AB, lot no. 200022).
- Each tube contained 50 µL of sample and 5 µL of antibody cocktail and was incubated according to standardized laboratory protocol.

Gating figure should show:

Sequential plots:

- FSC/SSC (debris exclusion)
- FSC-A vs FSC-H (doublet exclusion)
- CD45 gating
- CD3+ T cells
- CD38/CD138 plasma cells
- PD-1 / PD-L1 overlays

Doublet discrimination (FSC-A vs FSC-H) and exclusion of debris and non-cellular based on scatter characteristics were applied to all samples. Compensation was performed using controls according to standard flow cytometry compensation procedures and manufacturer’s instructions. Data analysis was conducted using a predefined gating template to ensure consistency across samples.

- T cells were defined as CD45+CD3+ events.
- PD-1 expression was quantified in gated CD3+ T cells as: Percentage of positive cells (%), median fluorescence intensity (MFI).
- Plasma cells were identified as CD38 bright/CD138+ events within the BM compartment.
- PD-L1 expression was assessed in gated plasma cells using both percentage positivity and MFI.

**
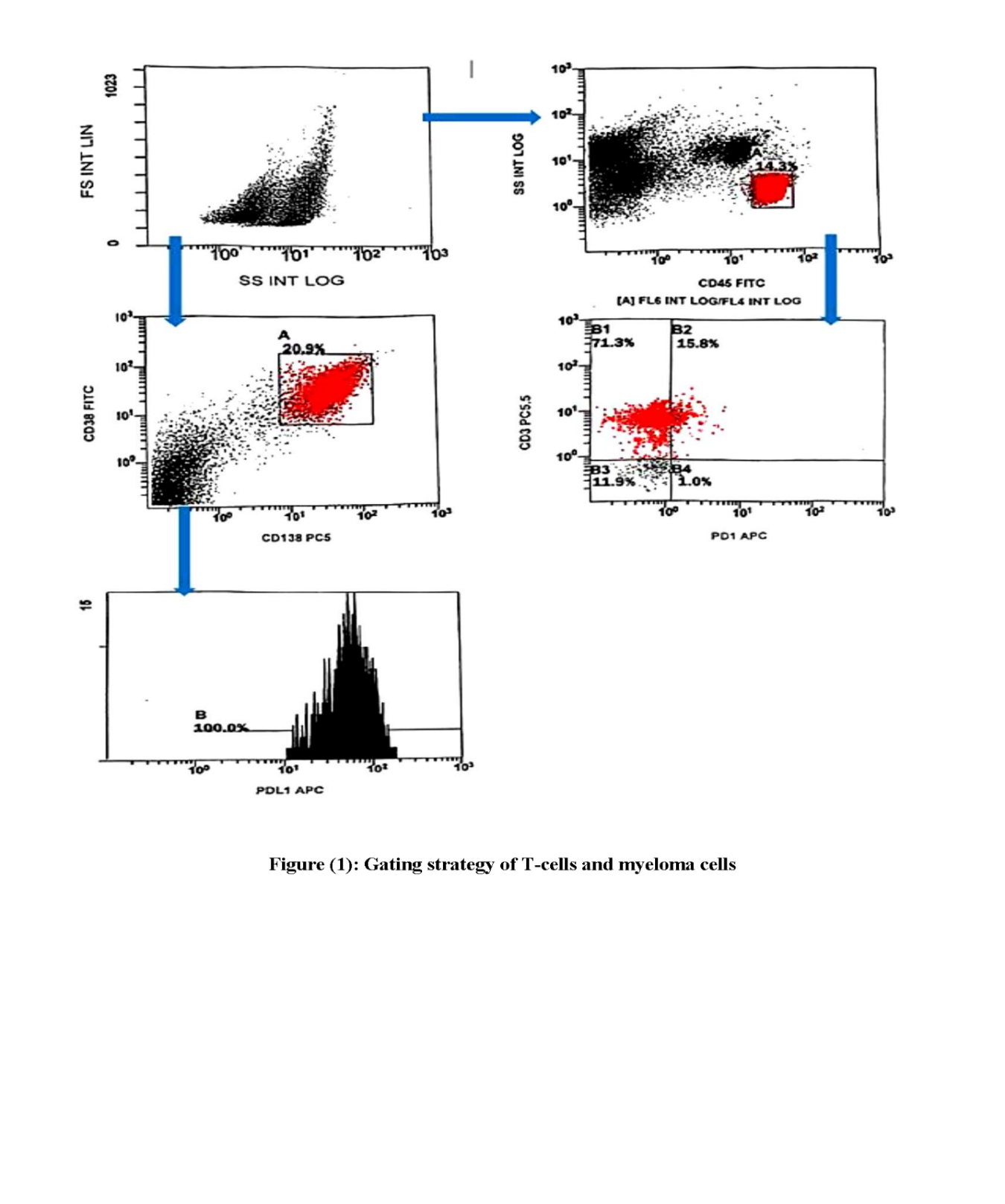
**

**Figure (S1): Flow cytometric strategy for evaluation of PD-1 in T cells and PD-L1 in plasma cells in multiple myeloma bone marrow samples.**
